# Resistance of cervical adenocarcinoma cells (HeLa) to venom from the scorpion *Centruroides limpidus limpidus*

**DOI:** 10.1186/1678-9199-19-20

**Published:** 2013-09-02

**Authors:** José María Eloy Contreras-Ortiz, Juan Carlos Vázquez-Chagoyán, José Simón Martínez-Castañeda, José Guillermo Estrada-Franco, José Esteban Aparicio-Burgos, Jorge Acosta-Dibarrat, Alberto Barbabosa-Pliego

**Affiliations:** 1 Centro de Investigación y Estudios Avanzados en Salud Animal (CIESA), Facultad de Medicina Veterinaria y Zootecnia (FMVZ), Universidad Autónoma del Estado de México (UAEM), Carretera Panamericana Toluca-Atlacomulco, km 15.5, código postal 50200, Toluca, México

**Keywords:** Scorpion venoms, Cytotoxic tests, Apoptosis, HeLa cells, Anticancer agents, *Centruroides* toxins

## Abstract

**Background:**

The venom of *Centruroides limpidus limpidus* (Cll) is a mixture of pharmacologically active principles. The most important of these are toxic proteins that interact both selectively and specifically with different cellular targets such as ion channels. Recently, anticancer properties of the venom from other scorpion species have been described. Studies *in vitro* have shown that scorpion venom induces cell death, inhibits proliferation and triggers the apoptotic pathway in different cancer cell lines. Herein, after treating human cervical adenocarcinoma (HeLa) cells with Cll crude venom, their cytotoxic activity and apoptosis induction were assessed.

**Results:**

Cll crude venom induced cell death in normal macrophages in a dose-dependent manner. However, through viability assays, HeLa cells showed high survival rates after exposure to Cll venom. Also, Cll venom did not induce apoptosis after performing ethidium bromide/acridine orange assays, nor was there any evidence of chromatin condensation or DNA fragmentation.

**Conclusions:**

Crude Cll venom exposure was not detrimental to HeLa cell cultures. This may be partially attributable to the absence of specific HeLa cell membrane targets for molecules present in the venom of *Centruroides limpidus limpidus.* Although these results might discourage additional studies exploring the potential of Cll venom to treat human papilloma cervical cancer, further research is required to explore positive effects of crude Cll venom on other cancer cell lines.

## Background

Scorpions and their venom have been used for centuries as medical treatments in traditional medicine in India, China, Africa and Cuba [1]. Scorpion venom has been used to treat pain, epilepsy, tetanus, subcutaneous nodules, and cancer [2-5]. It has been suggested that such properties may be partially explained by the presence of a variety of pharmacologically active substances, such as amino acids, inorganic salts, nucleotides, biogenic amines, lipids, enzymes and toxic proteins [6,7].

Despite huge efforts to find a cure for cancer, current treatments such as surgery, chemotherapy and radiotherapy still have not achieved satisfactory levels of protection or remission [1]. Moreover, existing treatments cause undesirable side effects and damage to healthy tissue surrounding tumors in patients under treatment [8]. Furthermore, it has been shown that tumor cells can develop resistance to some therapies [9].

Recently, it has been reported that some scorpion venoms inhibit the growth of various cancer cell lines via three distinct mechanisms: blocking specific ion channels, specifically binding to cancer cell membrane proteins, impairing their ability to invade, and activating intracellular pathways of apoptosis [10-13].

Cytotoxic, antiproliferative and apoptogenic effects have been demonstrated *in vitro*, by using crude venom of different scorpion species. MCF-7 (breast cancer) and SH-SY5Y (human neuroblastoma) cell lines treated with *Odontobuthus dorie* venom accelerated cell death and increased production of nitric oxide (NO) and reactive nitrogen intermediates (RNI), which are molecules related to mitochondrial depolarization and activation of caspase-3, present in apoptosis [14,15]. *Rophalurus junceus* venom exhibited remarkable cytotoxic effects in a non-dose-dependent manner on the mouse myeloma cell line P3-X63 [16]. The venom of *Heterometrus bengalensis* showed antiproliferative and apoptosis-inducing properties on U937 and K562 human leukemia cell lines, where chromatin condensation and DNA fragmentation were observed [3]. Additionally, *Buthus martensi* venom induced cell death by apoptosis, reducing the growth of U251-MG glioma cells probably by inhibition and modulation of several ion channels [17].

The anticancer therapeutic potential of the venoms from the several existent Mexican scorpion species has not been studied in detail. Considering the evolutionary relationship among scorpion species whose venoms have shown activity against cancer cell lines, we tested the hypothesis that *Centruroides limpidus limpidus* (Cll) venom produces therapeutic effects against cancer cells. In the present work, we evaluated the apoptotic and cytotoxic properties of Cll crude venom on human cervical adenocarcinoma (HeLa) cells.

## Methods

### Venom source

*Centruroides limpidus limpidus* specimens were collected in the municipality of Pilcaya, Guerrero, Mexico and transported to the animal housing facility of the Center for Research and Advanced Studies in Animal Health (Centro de Investigación y Estudios Avanzados en Salud Animal), from the School of Veterinary Medicine and Zootechny (Facultad de Medicina Veterinaria y Zootecnia), Autonomous University of Mexico State (Universidad Autónoma del Estado de México). *C. limpidus limpidus* were phenotypically identified at the species level with the use of keys, fed *ad libitum* and reared under standard insectary conditions (25°C, 75% RH) [18].

### Venom collection and preparation

The venom of 50 scorpions was obtained by electrical stimulation (10–20 V) of the telson, diluted in 500 μL of double distilled water, centrifuged at 14000 × g for ten minutes at 4°C, after which the supernatant was recovered [19]. Protein concentration was determined using the Bradford assay (BioRad, USA) and absorption was read at 595 nm. For cytotoxicity assays, the venom was used immediately after collection. To test bioactivity in mice, the venom was aliquoted and stored at −80°C until further use [10].

### Venom bioactivity test

To determine the bioactivity of Cll venom after collection, Balb/c mice (18 g, four weeks old, n = 9) were intramuscularly inoculated with 100 μL of 2.27 μg/μL of diluted crude venom. A control group (CTRL–) was injected with 100 μL of sterile, double-distilled water. The time of death and symptoms after inoculation were recorded. Experimental protocols were conducted according to the guidelines of the Bioethics Committee of the UAEM-FMVZ, the Official Mexican Standard (or *Norma Oficial Mexicana* – NOM-0062-ZOO-1999) technical specifications for the care and use of laboratory animals, as well as international standards [20,21].

### Cell culture

HeLa cells (ATCC: CCL-2) were grown to confluence in T25 flasks (Sarstedt, USA) with DMEM (Gibco Laboratories, USA) at 37°C, 5% CO_2_, 10% fetal bovine serum (FBS; Atlanta Biologicals, USA) and 1% penicillin-streptomycin (Gibco Laboratories, NY). Cell culture passages were performed by trypsinization when confluence reached 90% according to published protocols [22,23]. Briefly, the medium was removed and the confluent monolayer cells were washed with PBS (Gibco-BRL, USA); then cells were incubated with 1 mL of 0.25% trypsin (Gibco-BRL, USA) and further incubated for three minutes. Cells were then resuspended by pipetting, transferred to 15 mL conical tubes and centrifuged at 300 × g for five minutes. The supernatant was then discarded and cells were resuspended in DMEM 10% FBS. Cells were counted with a hemocytometer and viability was determined using Trypan blue [24].

### Cytotoxicity and viability (MTS) assays

Ten thousand cells were kept in 96 well plates for 24 hours in 10% FBS-supplemented DMEM. Then, the medium was replaced with 100 μL of MFS (FBS-free DMEM), after which four different doses of venom (50, 100, 200 and 400 μg/100 μL per well) were tested. Cyclophosphamide (Genoxal® Baxter Oncology, 400 μg/100 μL of medium) was used as positive control (CFF) and untreated cells as negative control (CTRL–). Three replicates per treatment were conducted. After incubation, the number of viable cells was estimated through a commercial MTS colorimetric assay (CellTiter 96® Aqueous One Solution kit, Promega, USA) according to the manufacturer’s instructions. Colorimetric reactions were measured with a spectrophotometer (Epoch, serial number 1209284) at 490 nm.

### Macrophage culture

To evaluate the effect of Cll venom on physiologically normal cells, venom toxicity in Balb/c adult mouse macrophages was tested. Animals were intraperitoneally inoculated with 1 mL of sodium thioglycolate. Four days later, the mice were killed humanely and cells were harvested by peritoneal lavage [25]. Briefly, the peritoneal cavity was injected with 5 mL of cold RPMI-1640 (Invitrogen, USA) and lightly massaged. The injected fluid was then aspirated and centrifuged for three minutes at 300 × g. Recovered cells were centrifuged and then washed three times (300 × g for three minutes, PBS). Washed cells were then inoculated into a 96-well plate at 1 × 10^6^ cells/well in 10% FBS-supplemented RPMI-1640. After incubation at 37°C for two hours in 5% CO_2_, the plate was washed twice with RPMI-1640 to remove non-adherent cells [26]. Adhered macrophages were exposed to 0 (CTR–), 50, 100 or 400 μg/100 μL of crude Cll venom in RPMI-1640 MFS. After a 24-hour incubation period, cell viability was determined according to the previous description.

### Apoptosis

To determine whether dying cells had undergone apoptosis, the ethidium bromide/acridine orange (EB-AO) staining method was adapted for the 96-well plate [24,27]. Briefly, 10^4^ cells/well were seeded in a 96 flat-bottomed well microtiter plate (Sarstedt, USA) containing 10% FBS-supplemented DMEM and incubated under controlled conditions (37°C and 5% CO_2_). After a 24-hour incubation period, the medium was replaced with 100 μL of fresh MFS, containing different venom concentrations, and further incubated for 24 hours. Then, the plate was centrifuged at 129 × g for five minutes and 8 μL of EB-AO was added to each well. Cells were observed under 40× magnification to search for typical apoptosis-induced morphological changes, such as apoptotic vesicles and chromatin condensation. Images of the cells were obtained with a Leica DMIL inverted fluorescence microscope equipped with a Nikon Coolpix S2500 digital camera.

## Results

### Venom bioactivity

**Figure 1:**
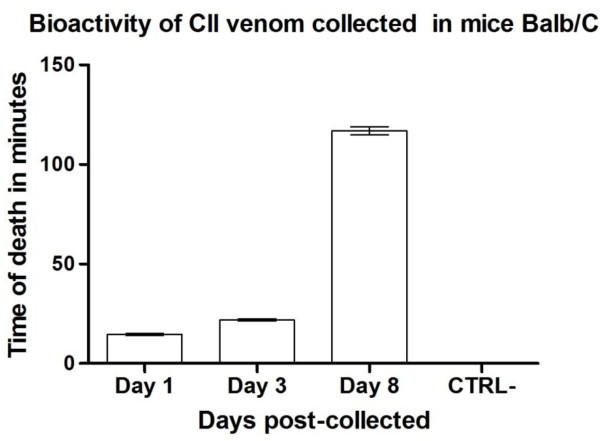
Venom bioactivity at different days post-collection differed statistically (p < 0.05) in four-week-old Balb/c mice among treatments despite a lower bioactivity tendency found in venom 8 days post-collection.

The mice inoculated with 100 μL of crude Cll venom at 2.27 μg/μL displayed ataxia, nasal pruritus, piloerection, tachypnea, tremor, seizures and excitability. As the time from venom collection to inoculation increased, venom-challenged individuals displayed longer survival, which suggests time-dependent venom degradation and loss of bioactivity after collection (Figure 1).

### Cell morphology

**Figure 2:**
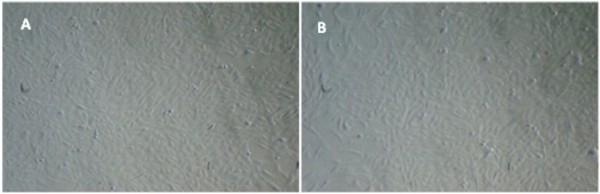
**Morphology of HeLa cells 24 hours after incubation with MFS: only one dose is shown since no differences were observed among different doses. (A)** Venom dose of 400 μg/100 μL and **(B)** untreated control cells.

HeLa cells were monitored 24 hours after exposure to 50, 100, 200 or 400 μg/100 μL of crude Cll venom. Treated cells did not show morphological changes, while the monolayer remained attached to the bottom of the plate. There was no evidence of membrane rupture or release of cytosolic contents in the culture medium and no morphological changes were apparent when compared to control cells (Figure 2A and B).

### Cell viability (MTS)

**Figure 3:**
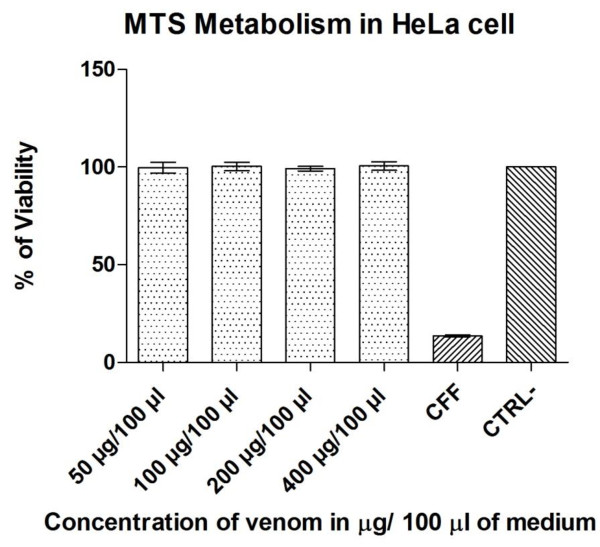
**MTS metabolism in Hela cells after treatment with different doses of venom (50-400 μg/100 μL of medium), positive control (CFF, cyclophosphamide 400 μg/100 μL) and negative control (CTRL–).** Optical density (OD) was read at 490 nm, the data were plotted according to the mean and SD. Treatment means differed significantly with respect to the positive control but not for the negative control (p < 0.05).

**Figure 4:**
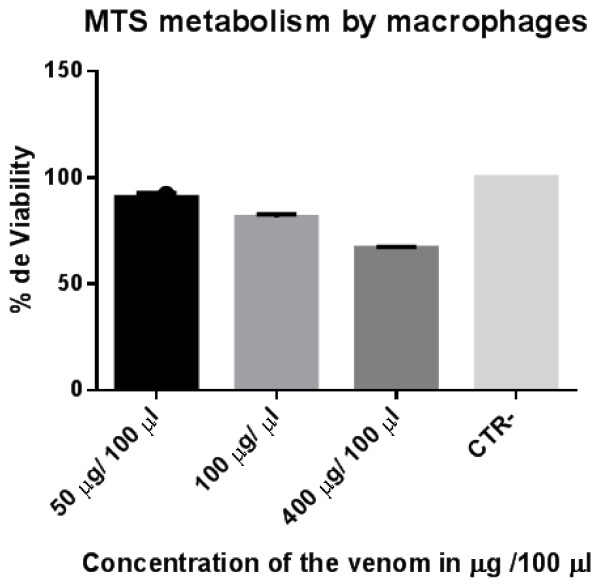
MTS metabolism by macrophages and viability percentage showed significant difference between treatment means at p < 0.05.

HeLa cells treated with doses ranging from 50–400 μg/100 μL of crude Cll venom showed viability of nearly 100%. However, cells treated with cyclophosphamide displayed ~80% mortality (Figure 3). In contrast, the same treatment induced cell death in Balb/c mice macrophages in a dose-dependent manner. Cell death was determined by evidence of necrosis (Figure 4).

### Apoptotic and necrotic effects

**Figure 5:**
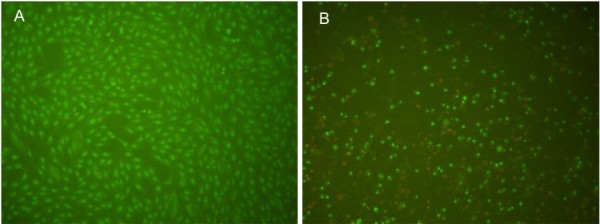
**Images showing Hela cells after treatment with scorpion venom. (A)** Cells treated with scorpion venom up to 400 μg/100 μL, and **(B)** positive controls showing apoptotic cells treated with cyclophosphamide (400 μg/100 μL).

Crude Cll venom cytotoxicity was also explored by assessing apoptosis. Post-venom-treated HeLa cells (24 hours) did not display typical apoptotic signs, such as nuclear condensation or DNA fragmentation (Figure 5A). Observation of intact nuclei also suggested lack of necrosis. However, cyclophosphamide-treated HeLa cells (positive control) showed typical apoptotic features such as chromatin condensation, monolayer loss, detachment from the plate, and cell shrinkage (Figure 5B).

## Discussion

It has been previously shown that exposure of several cancer cell lines to scorpion venoms induces major changes in cell morphology, loss of cell monolayers, cell contraction and death by either apoptosis or necrosis [14-17]. Our results showed that crude Cll venom-treated HeLa cells did not display any of the toxic effects produced by other scorpion species venom and cancer cell lines.

To find out whether bioactivity was lost after collection in a time-dependent manner, this parameter was evaluated at different time points after collection. Mice injected with 227 μg of crude Cll venom displayed the expected features of scorpionism, although onset of symptoms and death occurred within a shorter time [25]. This acceleration might be due to the fact that mice were challenged with a concentration higher than the lethal dose calculated for Cll (unpublished data). Although its bioactivity was reduced over time, mice mortality confirmed that venom remained bioactive even several days after collection (Figure 1).

MTS metabolism assays detected no venom-related effects on the viability of HeLa cells (Figure 3). These results contrast with what has been reported by others, specifically regarding the effects of crude venom of different scorpion species on several cancer cell lines, where cell viability is affected in a dose-dependent manner [4,14,15]. However, we found that crude Cll venom was toxic to Balb/c mice macrophages, as previously described in a *Tityus serrulatus* scorpion venom challenge of Balb/c mice macrophages [28]. Interestingly, Cll venom-induced macrophage mortality at 50 μg/mL was 9.3% (Figure 4), higher than that previously reported in *T. serrulatus* (5%).

Prior studies on the effect of arachnid venom and cytotoxicity on HeLa cells contrasted with our results. The venom of *Macrotele raven*, which induced cell death by apoptosis and cytotoxicity, was diluted in PBS [29]. Interestingly, cells incubated in PBS for 24 hours suffer osmotic shock and die due to either necrosis or apoptosis, or both [30]. To test the effect of PBS, we cultured HeLa cells in PBS and venom resuspended in PBS. We observed cell death under both treatments (data not shown). When venom was diluted in double-distilled water as in other protocols, we did not observe any toxic effects [10].

The most frequently reported cause of cell death induced by crude or fractionated scorpion venom is apoptosis, in which membrane receptors such as Fas-L have been shown to be involved. This can activate the extrinsic pathway of apoptosis and may also involve caspases 8, 9 and 3 [10]. It has also been reported that the venom elevates NO and RIN production, causes depolarization of the mitochondria, releases pro-apoptotic factors, and activates the intrinsic apoptosis pathway [14,15]. In the present study, cell death by apoptosis or lysis was not observed. However, apoptosis was induced in positive control cells when treated with the antineoplastic cyclophosphamide (Figure 3). Morphological changes such as cell shrinkage and loss of contact with other cells were also observed, whereas BE-AO staining revealed chromatin condensation (Figure 4), which is a typical feature of apoptosis [24,27].

The absence of a Cll-venom effect on HeLa cells could be partially explained by the lack of specific membrane targets, such as ion channel proteins, some of which are absent in some cancer cells, which regulate gene expression of ion channels according to their needs [31]. Much has been reported regarding the resistance of cancer cells to new drugs and treatments. However, one of the most important features involves overexpression of genes encoding proteins that block mechanisms and, in turn, switch on the apoptotic death pathway. It has been reported that HeLa cells over-express Bcl-2 and XIAP molecules, which confer resistance against apoptosis [32]. It has also been shown that down-regulation of XIAP sensitizes a cancer cell to apoptosis induced by anticancer drugs [33].

BAX is another protein that plays an important role in apoptosis. Human papilloma virus protein E6 degrades P53, and cannot activate the transcription of BAX [34]. In normal cells, BAX forms heterodimers with Bcl-2, resulting in viable cells. P53 stimulates transcription of BAX and forms homodimers that activate the cellular changes leading to apoptosis. In cancerous cells, where P53 is not functional, the level of BAX is not increased and as a result, cells do not start the apoptosis pathway [35]. It has also been shown that the use of ion channel blockers against K^+^ and Ca^2+^ may inhibit the process that triggers the pathways for cell death by apoptosis in some cell types [36]. Thus, it could be possible that Cll venom possesses ion channel blockers that prevent activation of the signaling pathway of the apoptotic cascade and stop cell death. It has been reported that loss of K^+^ ions causes depolarization of the membrane and forces entry of Ca^2+^ ions that participate in the activation of caspases and apoptosis [37].

Although our results demonstrate a toxic effect of Cll on macrophages and not on HeLa cells, it is possible that this venom is cytotoxic against cancer cells other than HeLa. Further studies of the effect of Cll venom on different cancer cell lines should be performed to assess its potential value as an anticancer agent.

## Conclusions

*Centruroides limpidus limpidus* venom showed no negative effects on HeLa cell cultures, possibly due to the absence of specific-HeLa cell membrane target molecules in the venom. We conclude that crude Cll venom is not a promising candidate for treating cervical uterine cancer. However, prospective studies on other cancer cell lines are needed to assess any anticancer properties in Cll venom.

### Ethics committee approval

The present study was approved by the Ethics Committee and Animal Welfare of School of Veterinary Medicine and Zootechny (Facultad de Medicina Veterinaria y Zootecnia), Autonomous University of Mexico State (Universidad Autónoma del Estado de México).

## Competing interests

The authors declare that there are no competing interests.

## Authors’ contributions

The author’s contribution is as follows: conceived and designed the experiments – BPA, VCJC, MCJS, COJME; performed the experiments – COJME, ABJE; analyzed the data – BPA, VCJC, MCJS, COJME; contributed reagents/materials/analysis tools: BPA, EFJG, VCJC; wrote and approved the paper: BPA, VCJC, MCJS, COJME, ADJ. All authors read and approved the final manuscript.
